# Asystolic Cardiac Arrest Associated With Unstable Bradycardia During Augmentation Mammaplasty: A Case Report

**DOI:** 10.1093/asjof/ojab047

**Published:** 2021-11-20

**Authors:** Nicole R Vingan, Steven Teitelbaum, Rita Moorman, Jeffrey M Kenkel

**Affiliations:** University of Texas Southwestern Medical Center, Dallas, TX, USA; associate clinical professor of plastic surgery, David Geffen School of Medicine at UCLA, Los Angeles, CA, USA; American Board of Anesthesiology in private practice in Santa Monica, CA, USA; Department of Plastic Surgery, University of Texas Southwestern Medical Center, Dallas, TX, USA

## Abstract

Cardiac arrest is a rare but reported complication during breast augmentation surgery. It is even more rare in a reportedly healthy patient without preexisting cardiac disease. The authors present the case of a healthy 34-year-old female who underwent elective bilateral augmentation mammaplasty and experienced unanticipated asystolic cardiac arrest intraoperatively following general anesthesia supplemented by a regional pectoral (pec I) nerve block. The performing plastic surgeon provided cardiopulmonary resuscitation while the anesthesiologist initiated a rescue protocol per Advanced Cardiac Life Support (ACLS) guidelines. Fortunately, the patient was resuscitated in a timely manner and had a successful return of spontaneous circulation within 1 minute. This case report serves to briefly review the literature and recommendations on proper resuscitation of cardiac arrest per ACLS protocols as well as discuss unstable bradycardia in otherwise healthy patients undergoing breast augmentation surgery. Plastic surgeons and anesthesiologists who perform this procedure should be aware of the possible, rare but serious progression to asystole as well as the proper resuscitative measures to take should they be required.

**Level of Evidence: 5:**

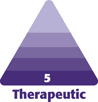

Per the 2020 Aesthetic Plastic Surgery National Databank Statistics Report, breast augmentation continues to be among the top 5 cosmetic surgical procedures, with 252,022 augmentation mammaplasties performed in 2020 alone.^1^ As this is an extremely common procedure that many plastic surgeons will routinely perform in their practice, a review of this topic and its risk profile is of utmost importance. Some of the possible risks and complications discussed with patients undergoing augmentation mammaplasty include bleeding/hematoma, infection, seroma, sensory changes to the nipple and breast, scarring, breast asymmetry, implant visibility/palpability, implant rippling, implant malposition, implant rupture, implant rotation, soft tissue stretching, glandular atrophy over time, galactorrhea, capsular contracture, anaplastic large cell lymphoma (related to implant), and the need for possible reoperation at some stage in the future.^2^ The aforementioned list does not account for intraoperative complications that may arise, such as the possibility of intraoperative cardiac arrest.

Risk factors that may increase perioperative morbidity include patient-specific health status, type of surgery, and length of surgery. Formal assessment of risk can be carried out through multiple metrics and serves an important function in estimating the individual patient’s surgical risk and guiding optimal perioperative management. One such risk assessment is the American Society of Anesthesiologists (ASA) Physical Status Classification System.^3^ Using this system, the following patient was noted to be ASA I, which is defined as a healthy patient. Although this grading schema cannot be the sole determinant of perioperative risk, it is still a helpful indicator when combined with other factors.^3^

A 2012 paper by Schusterman and Schusterman described 3 cases of profound intraoperative bradycardia during breast augmentation on healthy females. As stated in their report, bradycardia progressing to frank asystole is rare and is even more so in young, otherwise healthy patients undergoing aesthetic surgery.^4^ The current literature regarding perioperative hypotension and bradycardia progressing to asystole in this specific population is limited. This case report serves to address how seemingly innocuous bradycardia can degenerate rapidly into asystole and to remind plastic surgeons and anesthesiologists that cardiac arrest can happen even in low-risk situations. Thus, it is important to emphasize that surgery should only be performed in accredited operating rooms properly staffed with well-trained anesthesia staff, including board-certified anesthesiologists as well as certified registered nurse anesthetists, and operative personnel who can manage acute events such as the one described. Moreover, the importance of remaining up to date on Advanced Cardiac Life Support (ACLS) protocols and algorithms, for all individuals, including surgeons and nurses, working in the operating room, is imperative.

## CASE PRESENTATION

Regarding the following case presentation, written consent was provided, by which the patient agreed to the use and analysis of her data. A 34-year-old physically active female with no significant past medical history who had flown into town the day before surgery presented to an outpatient surgery center for elective bilateral augmentation mammaplasty. She had a baseline bradycardia of 51 beats per minute (bpm) upon arrival. The entirety of her preoperative workup, including bloodwork, electrocardiogram (ECG), health history, and physical examination, was normal, and she denied any cardiac history. The patient’s weight on the date of surgery was noted to be 62.7 kg. Anesthesia induction was completed with propofol, rocuronium, ondansetron, dexamethasone, promethazine, cefazolin, and fentanyl without complications, and bilateral pectoral nerve blocks with bupivacaine 0.25%, 20 mL each side, were given under ultrasound guidance. Anesthesia was maintained with sevoflurane. The surgery start time was approximately 15 minutes following the pectoral nerve blocks, but before incision, the patient’s heart rate dropped between 40 and 50 bpm while remaining in sinus rhythm. During this time, she remained normotensive with an oxygen saturation of 99%. An incision was made in the right inframammary fold and a retropectoral pocket was then successfully created on the right. Just after incision for the left retropectoral pocket, the patient experienced another decrease in her heart rate to 33 bpm followed by asystolic cardiac arrest. At this time, the performing plastic surgeon was informed, surgery was halted, and cardiopulmonary resuscitation (CPR) was initiated. CPR was halted to administer atropine; however, only slow escape beats were noted without a pulse, so CPR was resumed as per ACLS protocols. The performing surgeon provided CPR for less than 1 minute before return of spontaneous circulation (ROSC) following administration of intravenous (IV) epinephrine. After ROSC, the patient had rebound sinus tachycardia and hypertension, which was treated with esmolol. Paramedics were called and the patient’s incisions were closed, and she was transferred to a local hospital.

In the emergency department, the patient denied any chest pain or shortness of breath. Initial ECG demonstrated ST elevation in leads I and aVL, indicating lateral wall stress, with T wave inversion in the inferior leads. She had mildly elevated troponin and D dimer levels, while the rest of her labs were within normal limits, including a negative COVID-19 test. At this point, the patient had an uneventful recovery from the incident. Although initial history taken preoperatively was insignificant, she was interviewed again in the hospital and reiterated that there was no personal or family history of issues with anesthesia, premature cardiac disease, or sudden cardiac death. Imaging performed on the patient included echocardiography showing a structurally normal heart with normal left ventricular ejection fraction and computed tomography (CT) pulmonary angiogram negative for pulmonary embolism. Per cardiology, her results were normal. She was noted to be an athletic female with resting sinus bradycardia with no evidence of atrioventricular block assumed to represent high vagal tone. The patient remained asymptomatic in the hospital overnight. At this point in time, no additional inpatient treatment was deemed necessary for her, and the patient was discharged the following day with instructions to wear a Holter monitor and follow-up outpatient with a cardiologist.

Four months later, the patient underwent an uneventful breast augmentation in combination with laparoscopic inguinal hernia repair. In preparation for her second operation, the anesthesiology team reviewed previous records pertinent to the asystole event. The team was aware of the unstable nature of the patient’s bradycardia, and, therefore, it was monitored during the case due to the possibility of a predisposition for cardiovascular complications.

## DISCUSSION

Cardiac arrest during the perioperative period is a rare and unanticipated outcome during elective surgery of any kind; however, cardiovascular complications are particularly uncommon in aesthetic surgery, where patients are characteristically healthy. Reports of such complications in the literature, when they do appear, occur in patients with known comorbidities that predispose them to complications rather than in young asymptomatic patients with perioperative bradycardia.^4,5^ The latter occurrence, although limited in incidence, remains an important topic to be aware of. A review by Schusterman and Schusterman discusses 3 cases of spontaneous bradycardia progressing to asystole in patients very similar to the one discussed above. The patients in this report were all healthy, adult female patients with a history of endurance training and resting bradycardia. The authors discuss the plausibility that changes in the cardiovascular system of athletic individuals can predispose them to cardiac complications during surgery.^4,6^ Evidence suggests that resting bradycardia in humans following endurance training is largely attributable to high vagal tone.^7^ To echo the sentiment of the Schusterman and Schusterman report, it is reasonable to consider that athletic patients, such as the one discussed above, undergoing aesthetic surgery may experience an increase in parasympathetic tone relative to sympathetic tone predisposing them to profound bradycardia and even cardiac arrest. Surgeons and anesthesiologists need to be cognizant of the possibility of a sudden, rather than gradual, decrease in heart rate in patients with an underlying bradycardia and perhaps intervene with vagolytic agents such as glycopyrrolate and/or atropine sooner rather than later.

While the nature of this patient’s arrest points to overwhelming vagal tone, one should be aware of other differentials. Sudden and unanticipated cardiac arrests reported in literature pertain to spinal anesthesia and popliteal nerve blocks, but infrequently following pectoral nerve blocks.^8-14^ Pectoral nerve (Pecs) blocks I and II are regional anesthetic techniques aimed to anesthetize the lateral and medial pectoral nerves at the interfascial plane between the *pectoralis major* and *pectoralis minor* muscles, with Pecs II additionally aiming to block intercostal nerves 3-6, intercostobrachial branches, and the long thoracic nerve.^15-18^ There is a wide range of procedures in which the use of such blocks is beneficial, including insertion of breast expanders, mastectomies, sentinel node biopsies, axillary dissections, placements of ports, pacemakers, and implantable cardiac defibrillators and tumor resections.^15^ A recent randomized control trial by Aarab et al examined routine use of Pecs blocks for analgesia during aesthetic breast surgery. The trial investigated whether addition of the block to systemic multimodal analgesia would prove superior to analgesia alone for postoperative pain control following aesthetic breast surgery. At the conclusion of their study, Aarab et al were able to show that pectoral nerve blocks, in conjunction with multimodal pain medication regimens, were effective in alleviating perioperative pain after aesthetic breast surgery. Additionally, the combined use was also associated with reduced opioid consumption in the immediate postoperative period.^19^ To date, there are few published complications of Pecs blocks, mainly concerning risk of pneumothorax, local anesthetic systemic toxicity (LAST), iatrogenic vascular, or nerve injury or failure to anesthetize the desired area.^15,20^ Use of ultrasound guidance can help diminish the risk of complications, as can performing aspiration tests throughout administration. Toxicity from local anesthesia primarily affects the central nervous system and cardiovascular system.^21-24^ A review by Di Gregorio et al reported initial sympathetic activation as a common sign of cardiovascular toxicity, but bradycardia and hypotension can also occur. Toxicity can progress to arrhythmias, specifically ventricular tachycardia or fibrillation, and even asystole.^25^ In this situation, we do not believe that LAST caused the patient’s cardiovascular collapse due to atypical arrhythmia presentation and rapid ROSC without the use of lipid emulsion therapy, a current standard of care recommended by the American Society of Regional Anesthesia and Pain Medicine.^24,26-28^ Instead, we believe that the bilateral pectoral nerve blocks were a compounding variable on the pronounced vagal tone that blunted her pain to such an extent that the predominant response remained parasympathetic in nature.

This case brings to light the importance of proper management of acute intraoperative cardiac arrest. The treatment of evolving cardiac arrest is outlined in the Adult Cardiac Arrest Algorithm designed by the American Heart Association (AHA).^29^ ACLS guidelines developed by the AHA and European Resuscitation Council outline proper resuscitative protocols to use in these scenarios. The guidelines are reviewed continually and are periodically updated based on new and evolving literature.^30-32^ Although the occurrence of asystolic cardiac arrest in a healthy adult female with no known risk factors is rare, it is important to be aware of proper resuscitative measures and initiate proper protocols to avoid serious complications. This patient’s preoperative workup was considered unremarkable, and she was low risk based on multiple clinical factors, yet she ultimately required initiation of full ACLS protocol following cardiac arrest. The management of clinically significant bradycardia initially focuses on maintenance of airway and breathing. Atropine 0.5 mg IV, the dosing of which may be repeated until a total dose of 3 mg, is then administered as a vagolytic.^33^ In a situation where bradycardia degenerates into cardiac arrest, complete ACLS protocol is initiated as follows: CPR is begun and, depending on the rhythm, a shock or medication is provided, of which IV epinephrine 1 mg every 3-5 minutes is the first line. Reinitiation of CPR should commence following shock or medication administration. Constant monitoring of the patient’s rhythm should be performed to observe for continued administration of appropriate shocks or medications, including amiodarone or lidocaine, until ROSC.^29^ Thus, the importance of operating in an accredited operating room with ACLS-certified staff cannot be overlooked. Opportunities to improve knowledge and retention of ACLS protocols should be implemented regularly among operating room staff. Formal trainings and emergency drills can be utilized to provide hands-on practice in the management of arrest situations in accordance with proper guidelines. The benefit of running emergency drills can make the application of ACLS training more successful and can improve resuscitation outcomes following cardiac arrest. Secondarily, this case serves to educate the plastic surgery community on the clinical presentation and treatment of patients exhibiting possible local anesthetic systemic toxicity. Management focuses on early oxygenation and ventilation as well as swift initiation of ACLS protocols as discussed above. Following initiation of ACLS protocols, lipid emulsion therapy should be given. Intralipid therapy was first introduced as a treatment for LAST by Weinberg et al in 1998, and since its introduction, many case reports and studies have shown that the therapy can effectively be used to treat cardiovascular collapse caused by local anesthetics, of which bupivacaine-related cardiac arrest is particularly amenable.^27,34-36^ Guidelines for the use of lipid emulsion therapy for resuscitation purposes can be found through the American Society of Regional Anesthesia. In general, following initial airway management, lipid emulsion therapy is administered first with a large IV bolus of 20% lipid emulsion followed by continuous infusion for roughly 10 minutes following recovery of vital signs.^34,37,38^ Any facility utilizing local anesthesia for regional blocks or local infiltration should have 20% intralipid available to treat suspected cases of LAST.^39^

## CONCLUSION

This case report aims to remind the plastic surgery community that severe bradycardia progressing to asystole can occur in young, healthy patients with resting bradycardia. While most patients remain hemodynamically stable with low heart rates, the possibility of degeneration due to high vagal tone should be considered. Plastic surgeons and anesthesiologists are encouraged to stay keen that even seemingly the least risky cases with properly conducted operating room procedures can still result in unforeseen adverse events. In highlighting this risk of unstable bradycardia in these otherwise healthy aesthetic patients, we hope to make plastic surgeons who perform these cases more cognizant of the ensuing risks as well as the proper resuscitative measures to take, should they become necessary.
